# African Swine Fever Virus pD345L Suppresses JAK-STAT Signaling by Selectively Triggering STAT1 Degradation

**DOI:** 10.3390/ijms27115116

**Published:** 2026-06-05

**Authors:** Yingjia Gu, Meng Gao, Ying Huang, Chunhao Tao, Zhen Wang, Ruilong Xiao, Xinxin Jin, Hong Jia, Weifeng Yuan

**Affiliations:** Institute of Animal Sciences, Chinese Academy of Agricultural Sciences, Beijing 100193, China; 15231469612@163.com (Y.G.); 15090154589@163.com (M.G.); hy811cysbm@163.com (Y.H.); chunhao_tao@163.com (C.T.); wz2893963594@126.com (Z.W.); xiaoruilong1207@163.com (R.X.); 16622810205@163.com (X.J.)

**Keywords:** African swine fever virus, pD345L, innate immunity, JAK/STAT, immune evasion

## Abstract

African swine fever (ASF) is a highly lethal viral disease of pigs caused by the African swine fever virus (ASFV). The mortality rate is nearly 100%. Currently, it is known that the ASFV has a complex structure, and its genome encodes various immune escape proteins. However, the pathogenic mechanism of ASFV remains to be studied. This study found that ASFV pD345L significantly inhibits the activation of the ISRE promoter triggered by interferon (IFN) β and the production of downstream IFN-stimulated genes (ISG). We further reveal that pD345L may degrade STAT1 via the autophagy pathway and impede its nuclear translocation; this inhibitory effect is closely associated with its exonuclease activity. Our research results have clarified the impact of ASFV pD345L on the JAK/STAT signaling pathway, expanding our understanding of the inhibitory effect of ASFV-encoded proteins on the host’s innate immunity, and to some extent, contributing to the development of an African swine fever vaccine.

## 1. Introduction

African swine fever virus (ASFV) infection leads to the notorious African swine fever (ASF), which is a highly contagious viral disease of domestic and wild pigs [1]. The clinical symptoms induced by the virus infection may range from chronic or sub-clinical infection to peracute hemorrhagic disease, which depends on the viral virulence and age of the animals [2]. Generally, highly virulent strains can kill adult pigs within days [3]. The virus infection leads to great economic losses to the swine industry worldwide [4,5]. China reported its first outbreak in 2018, triggering unprecedented mortality in the national herd, crippling pork production, and posing a serious threat to ecological security [6,7]. Despite intensive research being performed worldwide, no safe and effective commercial vaccine is yet available.

ASFV is enveloped arbovirus virus, which is the only member of the family *Asfarviridae*. It completes replicative cycles in the cytoplasm. The double-stranded DNA viral genome ranges from 170 to 194 kb, encoding at least 168 proteins, including 68 structural and 100 non-structural (NS) proteins [8,9]. The NS proteins execute stage-specific functions throughout the viral life cycle, which are essential for the virus productive infection [10,11]. It preferentially infects cells of the monocyte–macrophage lineage [12,13]. Of note, these cells are the front-lines of defense against viral infection and are able to orchestrate both innate immunity and adaptive immune response [14]. The virus infection in these cells leads to dysfunction of these cells, which may consequently contribute to disease development. This macrophage tropism is therefore considered a central determinant of ASFV virulence [15], underscoring the importance of elucidating the mechanisms of how the virus disrupt host innate immune responses.

Type I interferon (IFN-I) is produced rapidly by infected cells. It orchestrates the early antiviral response [16]. The cascade begins when host pattern-recognition receptors detect conserved pathogen-associated molecular patterns, triggering signaling cascades that induce IFN-I transcription [17,18]. Secreted IFN-I then binds its heterodimeric receptor on the cell surface, activating Janus kinase 1 (JAK1) and tyrosine kinase 2 (TYK2). These kinases phosphorylate signal transducer and activator of transcription proteins 1 and 2 (STAT1/2), enabling their association with IFN-regulatory factor 9 (IRF9) to form the IFN-stimulated gene factor 3 (ISGF3) complex. Then the ISGF3 translocates to the nucleus, binds IFN-stimulated response elements (ISREs), and drives transcription of hundreds of IFN-stimulated genes (ISGs) that mobilize the antiviral state [19,20]. The JAK-STAT pathway is an essential signaling axis for immune regulation; virtually all interferons exert their antiviral and immunomodulatory effects by triggering this cascade [21].

It has been reported that ASFV encodes a series of immunomodulatory proteins that are capable of suppressing the host’s immune response for efficient replication [22,23]. For example, MGF100, MGF110, MGF300, MGF360, and MGF530/50 members of the virus’s multigene families, have been implicated in disabling innate immune responses [15,24]. ASFV H240R specifically disrupts the oligomerization of the adaptor protein STING, thereby effectively blocking the activation of the cGAS-STING signaling axis and inhibiting the production of type I interferon [25]. Concurrently, protein pH108R negatively regulates the innate immune response by suppressing NF-κB activation, thus attenuating host defense signaling [26]. Furthermore, protein pB318L exhibits a dual function in suppressing inflammatory responses; it not only inhibits NF-κB activation but also blocks the formation of the NLRP3 inflammasome, thereby comprehensively suppressing the release of inflammatory factors [27]. In the context of the RIG-I-like receptor pathway, protein pE248R employs a more complex inhibitory mechanism: it not only interacts with the Caspase Activation and Recruitment Domains (CARDs) of RIG-I to block its interaction with the mitochondrial adaptor MAVS but also binds to the Coiled-Coil Domain (CCD) of the E3 ubiquitin ligase TRIM25. This interaction impairs the multimerization capability of TRIM25, subsequently abolishing its ability to catalyze K63-linked ubiquitination of RIG-I and blocking the amplification of antiviral signaling cascades [28]. Collectively, these findings unveil a multilayered and multi-targeted immune evasion network orchestrated by ASFV.

The ASFV pD345L protein is a non-structural (NS) protein that functions as a lambda-like exonuclease with 5′–3′ exonuclease activity on single-stranded DNA substrates [29]. Recent studies have demonstrated that pD345L disrupts the cGAS-STING-mediated NF-κB signaling axis by interacting with IKKα/β and blocking their kinase activity [30]. To date, however, the impact of pD345L on the JAK-STAT signaling of the innate immune response has not been reported, leaving this an open question for future investigation. In this study, we examined the effects of the pD345L protein on the JAK/STAT pathway and elucidated the mechanisms underlying this interaction.

## 2. Results

### 2.1. ASFV pD345L Promotes Viral Infection Across Various Viruses Partially by Inhibiting IFN-β Signaling

**Figure 1:**
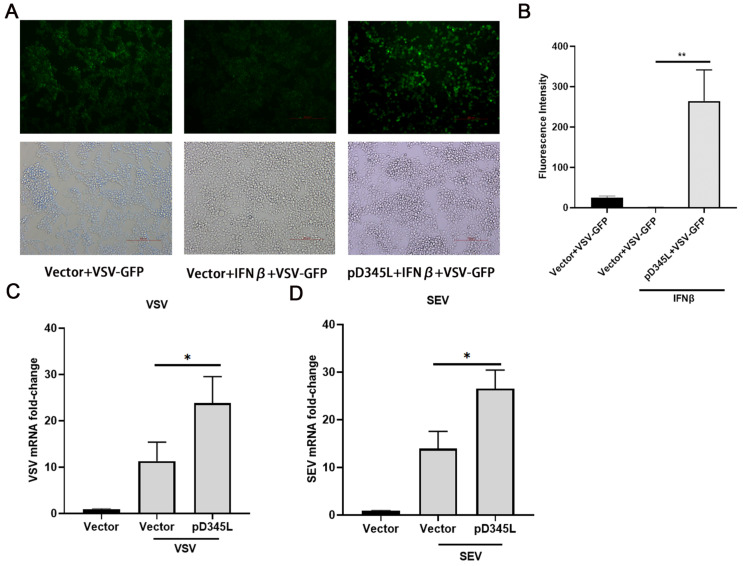
ASFV pD345L can promote viral infection. (**A**) HEK-293T cells were seeded in 24-well plates and cultured. Upon reaching the appropriate confluence, cells in each well were transfected with either an empty vector or the target plasmid. After 16 h of incubation, the cells were treated with or without IFN-β protein for 8 h, followed by infection with VSV-GFP at a multiplicity of infection (MOI) of 1. The cells were continuously monitored for 24 h post-infection. The upper panels show the green fluorescence channel, and the lower panels display the bright-field images. The green fluorescence indicates the expression of GFP, representing successful VSV infection and replication in the cells. (**B**) The fluorescence density statistical results of the corresponding fluorescence images in (**A**), bar = 200 μm.* *p* < 0.05; ** *p* < 0.01. The data were expressed as the mean ± SD value of the three experiments. (**C**,**D**) HEK293T cells were infected with VSV-GFP or SEV-GFP (MOI = 1) for 12 h, and then the viral mRNA levels were detected.

To evaluate the ability of pD345L to antagonize host innate immunity, HEK293T cells transfected with pD345L or an empty vector were infected with VSV-GFP and treated with IFN-β. As expected, IFN-β treatment resulted in a significant decrease in fluorescence intensity relative to the mock control, indicative of blocked productive infection (Figure 1A), and significantly stronger GFP signals was observed in the cells transfected with plasmid expressing pD345L-proteins (Figure 1A,B). Quantitative analysis indicated that the fluorescence intensity was increased to the control with transfection of blank plasmid. qRT-PCR analysis indicated that the transfection of pD345L plasmid leads to an increased infection of both VSV and SEV. The viral mRNA levels increased to approximately 2-fold, relative to the control (Figure 1C,D). Therefore, ASFV pD345L has the potential to promote viral productive infection partially via antagonizing the antiviral effects of IFN-β.

**Figure 2:**
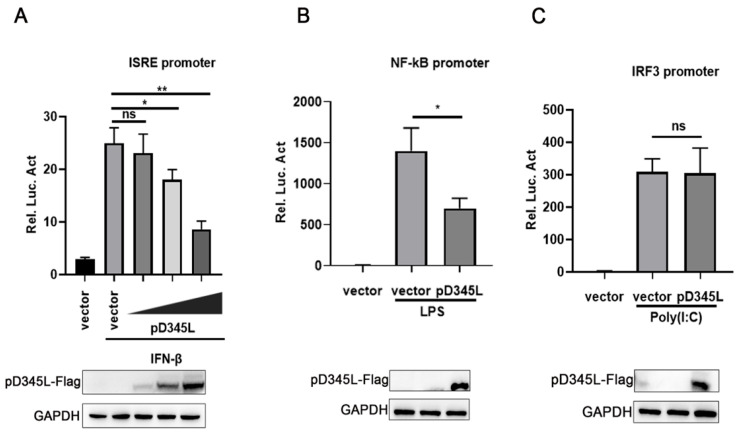
ASFV pD345L inhibits IFN-β signal transduction. (**A**) HEK-293T cells were inoculated and cultured in 24-well plates. After the cells reached an appropriate density, each well was transfected with an empty vector or different doses of the target plasmid (100, 200, 500 ng), pRL-TK plasmid (25 ng), and ISRE-Luc plasmid (125 ng), respectively. The samples were treated with or without IFN-β protein (1000 U/mL) 8 h before sample collection, and then luciferase determination was performed. (**B**) A suitable amount of pD345L plasmid, pRL-TK plasmid (25 ng) and NF-κB -Luc plasmid (125 ng) were transfected into HEK-293T cells. Eight hours before sample collection, a stimulant was added to the samples, and then luciferase assay was performed. (**C**) A suitable amount of pD345L plasmid, pRL-TK plasmid (25 ng), and IRF3-Luc plasmid (125 ng) were transfected into HEK-293T cells. Eight hours before sample collection, a stimulant was added to the samples, and then luciferase assay was performed. * *p* < 0.05; ** *p* < 0.01. The data were presented as the mean ± SD values of three independent experiments.

To further define how ASFV pD345L affects IFN-β signaling, HEK293T cells were co-transfected with an ISRE-luciferase reporter and increasing amounts of pD345L expression plasmid. Luciferase assays revealed that pD345L suppressed IFN-β-driven ISRE activation in a dose-dependent manner (Figure 2A). We next examined the effect of pD345L on IRF3- and NF-κB-mediated transcription. Cells were transfected with pD345L together with IRF3- or NF-κB-luciferase reporters and stimulated with the appropriate agonists (Figure 2B). pD345L did not reduce poly(I:C)-induced IRF3-luc activity (Figure 2B) but partially inhibited NF-κB-luc activation (Figure 2C), consistent with the findings as described elsewhere [30]. These findings suggested that ASFV pD345L has the potential disrupt IFN-β-stimulated signaling.

### 2.2. ASFV pD345L Inhibits IFN-β-Induced ISG Transcription

**Figure 3:**
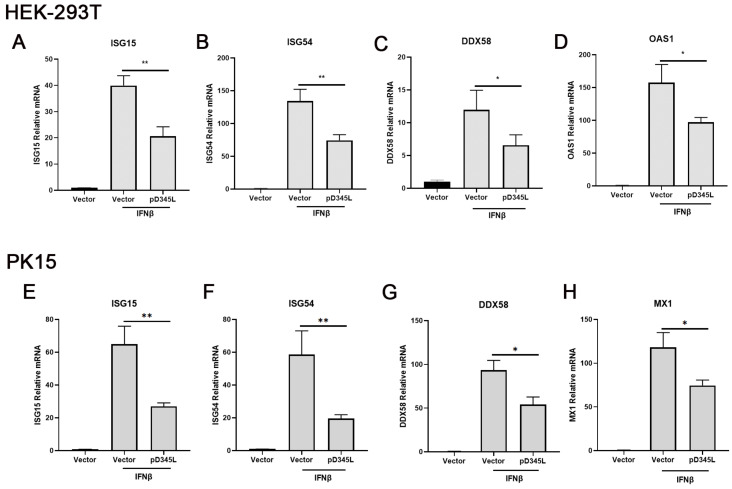
ASFV D345L inhibits the transcriptional level of interferon-stimulated genes. Transfected HEK-293T cells (**A**–**D**) or PK-15 cells (**E**–**H**) cultured in 24-well plates with pD345L expression plasmid (400 ng) or empty plasmid. After 24 h, the cells were treated with IFN-β (1000 U/mL) for 8 h. The mRNA transcriptional levels of ISG15 and others were detected by RT-qPCR. * *p* < 0.05; ** *p* < 0.01. The data were expressed as the mean ± SD value of the three experiments.

To rigorously assess whether pD345L antagonizes IFN-β signaling, HEK-293T and PK-15 cells were transfected with a pD345L-expressing plasmid and subsequently stimulated with IFN-β. RT-qPCR quantification of prototypical interferon-stimulated genes revealed that pD345L markedly suppressed transcription of IFN-β-induced ISGs (Figure 3). These results confirm that pD345L acts as an inhibitor of IFN-β signaling.

### 2.3. ASFV pD345L Disrupts ISGF3-Mediated ISRE Promoter Activation

**Figure 4:**
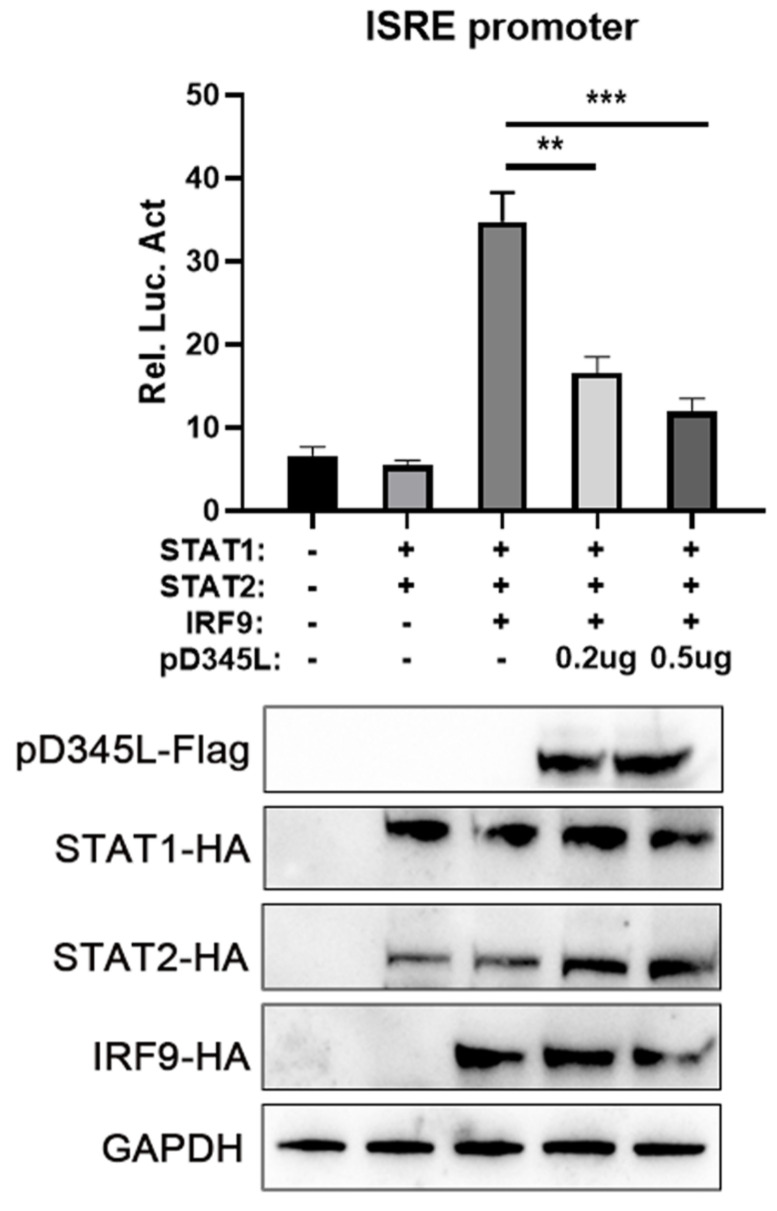
ASFV D345L inhibits the ISRE promoter activity induced by ISGF3. Different concentrations of Flag-pD345 L were used to express plasmids (0, 200 ng or 500 ng/well), as well as HA-STAT1 (400 ng/well), HA-STAT2 (400 ng/well), HA-IRF9 (400 ng/well), and ISRE−Luc Plasmids (100 ng/well), and pRL−TK plasmids (20 ng/well) were transfected into HEK-293T cells cultured in 24−well plates. After 28 h, the luciferase assay was conducted. ** *p* < 0.01 *** *p* < 0.001. The data were presented as the mean ± SD values of three independent experiments. + and − indicate the presence and absence of the treatment, respectively.

In the canonical JAK/STAT cascade, IFN-β triggers phosphorylation of STAT1 and STAT2, enabling their association with IRF9 to assemble the heterotrimeric transcription factor ISGF3. Once imported into the nucleus, ISGF3 binds ISRE elements and drives robust ISG transcription [31]. Even non-phosphorylated STAT1/2 and IRF9 can form an alternative “U-ISGF3” complex that similarly amplifies ISRE activity when present at high levels [32,33]. To test whether pD345L interferes with this axis, HEK-293T cells were co-transfected with fixed amounts of STAT1, STAT2, IRF9, and an ISRE-luciferase reporter, together with increasing doses of pD345L or empty vector. As a result, ISGF3 strongly activated the ISRE promoter, whereas ASFV pD345L suppressed this activation in a dose-dependent manner (Figure 4). The probed proteins of pD345L, STAT1/2, and IRF9 by Western blot validated the findings that pD345L targets the ISGF3 complex to antagonize IFN-β signaling.

### 2.4. ASFV pD345L Potentially Induces STAT1 Degradation Through the Autophagy Pathway

**Figure 5:**
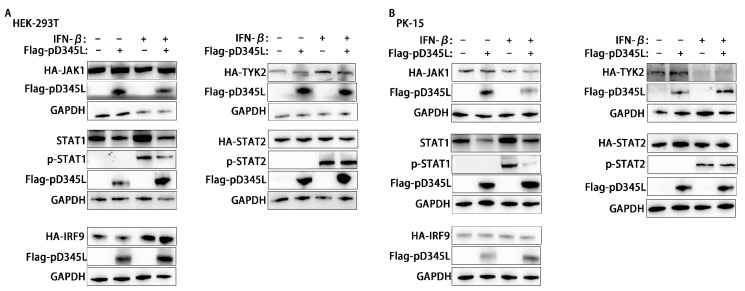
ASFV pD345L down-regulate the expression of STAT1 protein. HEK-293T (**A**) or PK-15 cells (**B**) were transfected with empty vectors or Flag-pD345L (800 ng) and plasmids at key sites of the JAK/STAT pathway (800 ng) and incubated for 24 h. Then IFN-β (1000 U/mL) treatment was carried out or not for 8 h. Cell lysates were analyzed by Western blotting using the specified antibodies. + and − indicate the presence and absence of the treatment, respectively.

The down-regulation of signal transduction molecules activated by IFN is a commonly used defense mechanism in many viruses. To dissect how the viral protein pD345L antagonizes IFN-β signaling, we examined its impact on the JAK/STAT cascade. HEK-293T and PK-15 cells were transfected with the pD345L expression plasmid, and the effect of pD345L on the proteins in the JAK/STAT pathway was evaluated by Western blotting. pD345L. Regardless of whether IFN-β treatment was carried out or not, pD345L significantly reduced the protein level of STAT1 but had no effect on other proteins in this pathway (Figure 5A,B). The experimental results show that the expression of pD345L protein will reduce the level of STAT1.

**Figure 6:**
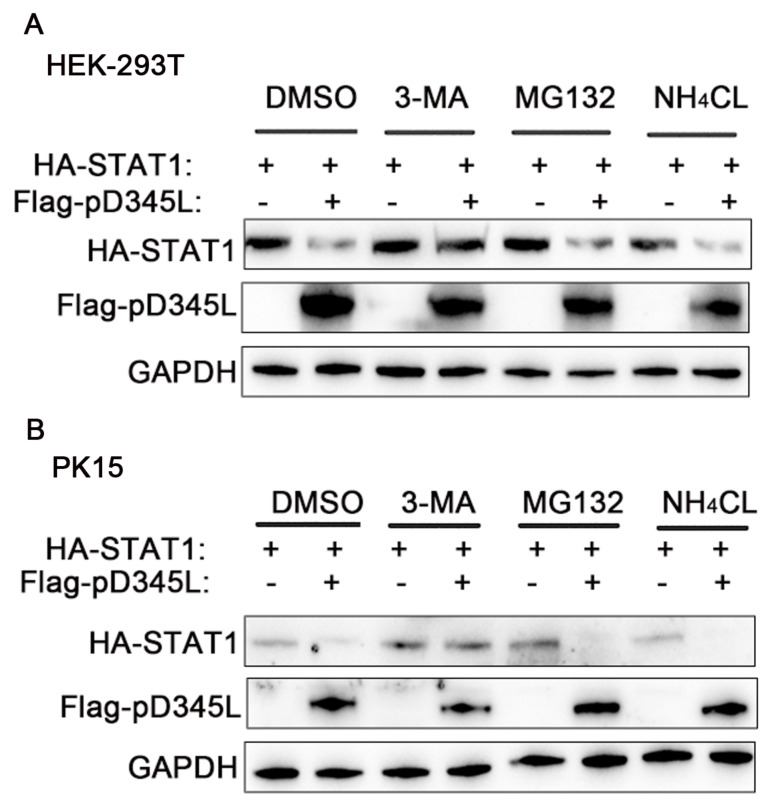
The effect of the inhibitor on the reduction in STAT1 mediated by pD345L. HEK-293T cells (**A**) or PK-15 cells (**B**) were transfected with the Flag-pD345L expression plasmid (800 ng/well) and the HA-STAT1 plasmid (800 ng/well). Eighteen hours after transfection, the cells were treated with IFN-β (1000 U/mL) and the specified inhibitors, namely 3-MA (500 ng/mL), MG132 (50 μM), NH_4_Cl (20 mM), for 6 h, and then Western blotting was performed. DMSO (2 μL/ well) was used as the blank control of the inhibitor. + and − indicate the presence and absence of the treatment, respectively.

To reveal the mechanism of why pD345L destabilize the protein levels of STAT1, pD345L transfected cells were treated with the canonical autophagosome inhibitor 3-MA, proteasome inhibitor MG132, and lysosomal inhibitor NH_4_Cl, respectively. We found that the degradation of STAT1 mediated by pD345L was inhibited by the autophagosome inhibitor 3-MA, but not by either proteasome inhibitor MG132 or the lysosomal inhibitor NH_4_Cl (Figure 6A,B). These results suggest that pD345L may degrade STAT1 via the autophagy pathway.

### 2.5. ASFV D345L Interacts with STAT1 and Interrupts IFN-I-Induced Nuclear Accumulation of STAT1

**Figure 7:**
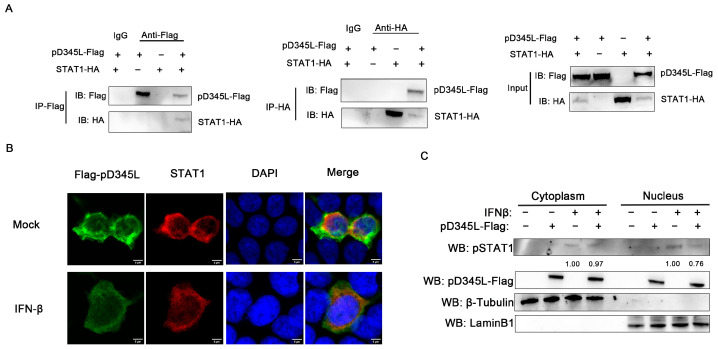
ASFV D345L interacts with STAT1. (**A**) D345L interacts with STAT1. HEK-293T cells were transfected with the expression plasmids Flag-D345L (3 μg) and HA-STAT1 (3 μg). After 24 h, the cells were treated with IFN-β (1000 U/mL) for 8 h and then co-immunized with the anti-flag antibody. The presence of STAT1 protein was analyzed by Western blotting with anti-HA antibody. (**B**) D345L is co-located with STAT1. HEK-293T cells were transfected with the expression plasmids Flag-D345L (2 μg) and HA-STAT1 (2 μg). After 24 h, the cells were treated with IFN-β (1000 U/mL) for 8 h and then observed. Nuclei were stained with DAPI (blue). Flag-pD345L and HA-STAT1 were visualized using Alexa Fluor 594 (red) and Alexa Fluor 488 (green), respectively. (**C**) ASFV pD345L hinders the nuclear translocation of STAT1.HEK-293T cells were transfected with pD345L plasmid or empty vector control and HA-STAT1 plasmid for 24 h. After treatment with IFN-β (1000 U/mL) for 8 h, the nuclear and cytoplasmic components were separated using the Minute nuclear and cytoplasmic extraction reagent, and then Western blotting analysis was performed with the relevant antibodies. + and − indicate the presence and absence of the treatment, respectively.

To determine the mechanism of how the viral protein pD345L antagonizes STAT1 signaling, we first examined their physical association. HEK-293T cells were transfected with pD345L or empty vector and analyzed by immunoprecipitation (IP) and immunofluorescence (IFA) assay. As a result, IP assay indicated that pD345L specifically associates with STAT1 (Figure 7A). HEK-293T cells were transfected with the Flag-pD345L and HA-STAT1 expression plasmids, and the localization of pD345L and STAT1 proteins was observed under a confocal microscope. In the absence of IFN-β treatment, pD345L and STAT1 proteins were colocalized in the cytoplasm. After IFN-β treatment, the STAT1 proteins in the cytoplasm were still colocalized with pD345L (Figure 7B). Thus, these two independent approaches establish that ASFV pD345L may associate with STAT1 in the cytoplasm.

It has been reported that nuclear import of phospho-STAT1 is essential for IFN-driven transcriptional responses [34]. We tested whether the viral protein pD345L has effects on STAT1 nuclear translocation. HEK-293T cells were transfected with pD345L or empty vector for 24 h, followed by stimulated without or with IFN-β for 8 h. Then, the fractions of either nucleus or cytoplasm were isolated using a commercial nuclear isolation kit and subjected to Western blot. Under the stimulation of IFNβ, Phospho-STAT1 was present in both nuclear and cytoplasmic fractions, but its nuclear abundance was markedly reduced in pD345L-transfected cells compared with mock controls (Figure 7C). Thus, pD345L has capacity to inhibit cytoplasm–nucleus translocation of Phospho-STAT1.

### 2.6. The Exonuclease Catalytic Site of ASFV pD345L Plays a Critical Role in Inhibiting the IFN-β Signaling Pathway

**Figure 8:**
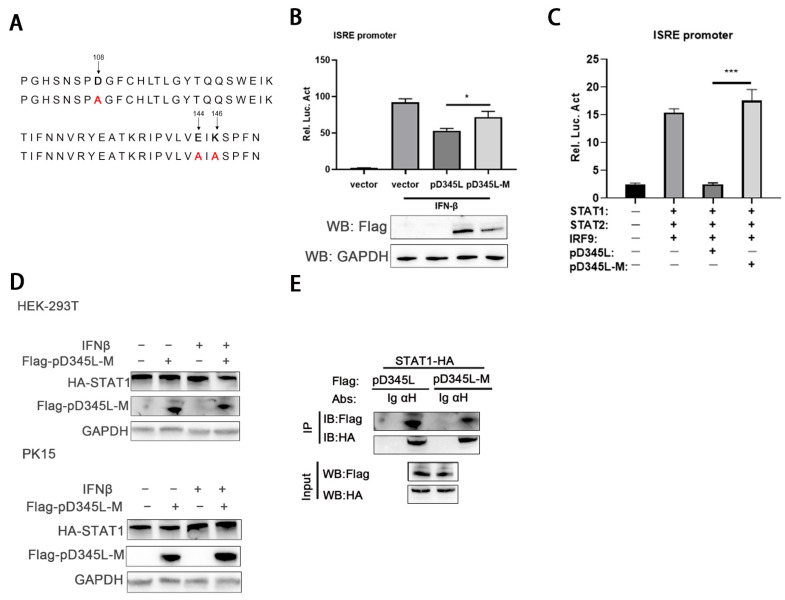
The inhibition of JAK/STAT signaling mediated by pD345L is related to its exonuclease activity. (**A**) Schematic diagram of the catalytically active mutant D345L (D345L-M). (**B**) On HEK-293T cells, empty vectors, D345L or D345L-M (400 ng), pRL-TK plasmids (25 ng), and ISRE-Luc plasmids (125 ng) were transfected, respectively. After 24 h of culture, samples were collected. Eight hours before collection, IFN-β (1000 U/mL) protein treatment was added or not added. Then luciferase determination was carried out. * *p* < 0.05, The data were expressed as the mean ± SD value of the three experiments. (**C**) On HEK-293T cells, transfect the empty vector, D345L or D345L-M (400 ng), HA-STAT1 (400 ng/well), HA-STAT2 (400 ng/well), HA-IRF9 (400 ng/well), ISRE-Luc plasmid (100 ng/well), and PR-TK, respectively. Plasmid (20 ng/well), samples were collected after 24 h of culture. IFN-β (1000 U/mL) protein treatment was added or not added 8 h before collection, and then luciferase determination was performed. + and − indicate the presence and absence of the treatment, respectively. *** *p* < 0.001, The data were expressed as the mean ± SD value of the three experiments. (**D**) ASFV pD345L-M does not affect the expression of STAT1 protein. HEK-293T or PK-15 cells were transfected with empty vectors or Flag-pD345L-M (800 ng) and HA-STAT1 (800 ng) and incubated for 24 h. Then IFN-β treatment (1000 U/mL) was carried out or not for 8 h. Cell lysates were analyzed by Western blotting using the specified antibodies. (**E**) HEK-293T cells were transfected with the expression plasmids Flag-D345L-M (3 μg) or Flag-D345L (3 μg) and HA-STAT1 (3 μg). After 24 h, the cells were treated with IFN-β (1000 U/mL) for 8 h and then co-immunized with the anti-flag antibody. The presence of STAT1 protein was analyzed by Western blotting with anti-HA antibody.

The pD345L protein contains an N-terminal 5′–3′ exonuclease domain that shows high similarity to the lambda phage exonuclease [30]. In this study, a catalytic mutant of pD345L (designated pD345L-M) was generated as illustrated in Figure 8A. Briefly, based on the catalytic center residues of the lambda phage exonuclease [35], aspartic acid 108 (D108), glutamic acid 144 (E144), and lysine 146 (K146) were substituted with alanine (A) in pD345L. These mutations were designed to disrupt its exonuclease activity. The mutant plasmid pD345L-M was used to assess its effect on ISRE activity using a dual-luciferase reporter assay. Our results showed that pD345L-M failed to suppress ISRE promoter activation (Figure 8B,C), a finding supported by detectable expression of the mutant protein. Consistently, pD345L-M also lost its ability to promote STAT1 degradation, as observed in both HEK-293T and PK-15 cells (Figure 8D). Furthermore, co-immunoprecipitation assays showed that pD345L-M retained its ability to bind STAT1 in transfected HEK-293T cells (Figure 8E). These results suggest that although the pD345L-M protein lacks exonuclease activity, it is still capable of binding to STAT1 but fails to induce its degradation. This finding, to some extent, explains why the suppression of the JAK/STAT signaling pathway is alleviated.

## 3. Discussion

The innate immune system serves as the primary barrier against viral infections. ASFV has evolved a sophisticated arsenal of proteins to counteract host defenses, ensuring its survival and propagation. In this study, we identified the ASFV-encoded nuclease pD345L as a potent negative regulator of the JAK/STAT signaling pathway. We demonstrate that pD345L suppresses IFN-β-induced antiviral responses by targeting STAT1 for degradation via the autophagy pathway and inhibiting its nuclear accumulation (Figure 1, Figure 2, Figure 3, Figure 4, Figure 5, Figure 6, Figure 7 and Figure 8). These findings uncover a novel mechanism of immune evasion employed by ASFV and highlight the multifaceted strategies viruses use to dismantle host immunity.

The JAK/STAT pathway is central to the establishment of an antiviral state following type I interferon (IFN) stimulation. Previous studies have documented several ASFV proteins that disrupt this pathway at distinct nodes [36]. For instance, MGF360-9L promotes the proteasomal degradation of STAT1 and STAT2 [37]; I7L blocks STAT1 phosphorylation [38]; MGF360-10L ubiquitinates and degrades JAK1 [39]; MGF360-12L and I215L deplete IRF9 [40,41]. Our characterization of pD345L reveals a mechanism that is distinct from these known regulators in several critical aspects. First, regarding the degradation machinery, while MGF360-9L relies on the ubiquitin-proteasome system, pD345L potentially mediates STAT1 turnover through the autophagy pathway. This suggests that ASFV exploits diverse cellular protein homeostasis systems to fine-tune the levels of key immune effectors. Second, regarding the molecular mechanism, the ability of pD345L to degrade STAT1 is closely associated with its exonuclease catalytic site. We identified aspartic acid 108 (D108), glutamic acid 144 (E144), and lysine 146 (K146) as critical residues mediating the degradation of STAT1 by pD345L. Such an enzyme activity-dependent interaction with a host transcription factor is relatively rare among known ASFV immunomodulatory proteins. Third, in terms of regulatory scope, pD345L not only reduces total STAT1 protein levels but also actively impedes the nuclear translocation of the remaining pool. This dual-layered inhibition—targeting both protein stability and subcellular localization—effectively prevents the assembly of the ISGF3 complex and the subsequent transcription of interferon-stimulated genes (ISGs), thereby ensuring a robust blockade of the antiviral response.

Beyond its effect on the JAK/STAT pathway, emerging evidence suggests that pD345L may also modulate the NF-κB signaling axis. The NF-κB and JAK/STAT pathways are not isolated entities but engage in extensive crosstalk to coordinate innate immune responses. A viral protein capable of simultaneously suppressing both pathways would exert a synergistic immunosuppressive effect: inhibiting NF-κB would dampen the initial production of pro-inflammatory cytokines and type I IFNs, while blocking JAK/STAT would prevent the amplification of the antiviral signal downstream of the IFN receptor. This “double-hit” strategy likely creates a highly permissive environment for early viral replication and systemic spread. Future investigations dissecting how pD345L balances the inhibition of these two interconnected pathways will be crucial for a comprehensive understanding of ASFV pathogenesis and its high virulence.

Given the pivotal role of pD345L in immune evasion, gene deletion represents a logical strategy for developing attenuated ASFV vaccines. Indeed, deletion of various non-essential genes has yielded promising vaccine candidates. However, our attempts to generate a viable ASFV mutant lacking the D345L gene were unsuccessful, suggesting that this gene is essential for viral replication in vitro or is indispensable for counteracting lethal host responses during the early stages of infection. This essentiality poses a significant challenge for traditional live-attenuated vaccine approaches targeting this specific gene. To overcome this hurdle, the development of a complementary cell line stably expressing pD345L appears necessary to rescue and propagate D345L-deleted mutants. Such a system would allow for the evaluation of the mutant’s immunogenicity and protective efficacy, potentially unlocking a new avenue for vaccine design.

We acknowledge certain limitations in our study. First, while pharmacological assays using 3-MA suggest that pD345L-mediated STAT1 degradation involves the autophagy pathway, these findings require further validation through genetic approaches (e.g., ATG5/7 knockout) to rule out off-target effects. Second, although catalytically inactive mutants indicate that pD345L’s exonuclease activity is critical for STAT1 degradation, we cannot entirely exclude the potential influence of mutation-induced conformational changes. Future structural biology studies and the use of specific small-molecule inhibitors will be essential to definitively corroborate the enzyme activity-dependent mechanism. At last, we acknowledge that PK-15 cells do not fully recapitulate the physiological environment of porcine alveolar macrophages (PAMs), the primary natural targets of ASFV. In future studies, we aim to overcome the technical challenges of primary cell cultures and validate the pD345L-mediated immune evasion mechanisms in PAMs or organoid systems. These efforts will further strengthen the physiological relevance of our conclusions.

## 4. Materials and Methods

### 4.1. Cell and Virus

Human embryonic kidney 293T (HEK293T) and porcine kidney 15 (PK-15) cells were obtained from Type Culture Collection of Chinese Academy of Science and cultured in Dulbecco’s modified Eagle’s medium (DMEM, Gibco, Grand Island, NY, USA) with 10% fetal bovine serum (FBS, Gibco), 100 U/mL penicillin (Gibco), and 100 µg/mL streptomycin (Gibco) at 37 °C in 5% CO_2_. Vesicular Stomatitis Virus (VSV-GFP) and Sendai Virus (SEV-GFP) were stored in the laboratory; they were tagged with GFP and stably express green fluorescence.

### 4.2. Antibodies and Reagents

Monoclonal rabbit anti-hemagglutinin (HA), anti-glyceraldehyde 3-phosphate dehydrogenase (GAPDH), and horseradish peroxidase-conjugated goat anti-rabbit IgG were purchased from Cell Signaling Technology (Danvers, MA, USA). Mouse monoclonal anti-flag were obtained from Solarbio (Beijing, China). Rabbit IgG and mouse IgG was supplied by Beyotime (Beijing, China). Anti-Phospho-STAT1 (Tyr 701) (Cat No. 28979-1-AP) and anti-STAT1 (Cat No. 10144-2-AP) were purchased from Proteintech (Rosemont, IL, USA). β-tubulin rabbit mAb and Anti-Phospho-STAT2 (Tyr 690) were obtained from Zhengnengbio (Beijing, China). Alexa Fluor 594-conjugated goat anti-rabbit IgG antibody and Alexa Fluor 488-conjugated goat anti-mouse IgG antibody were purchased from ZSGB-BIO (Beijing, China). Lipofectamine 3000 transfection reagent and double-luciferase reporter assay kits were obtained from InvivoGen (Hong Kong, China) and TransGen (Beijing, China), respectively.

Proteasome inhibitor MG132, lysosome inhibitor ammonium chloride (NH_4_Cl), autophagosome inhibitor 3-methyladenine (3-MA), and IFN-beta Protein were purchased from MedChemExpress (Monmouth Junction, NJ, USA). Dimethyl sulfoxide (DMSO) was supplied by Sigma-Aldrich (St. Louis, MO, USA).

### 4.3. Plasmid

The full-length sequence of African swine fever virus D345L with Flag tagged was synthesized by GenScript (Nanjing, China) and cloned into the p3 × Flag-CMV-7.1 vector. Hemagglutinin (HA)-tagged expression plasmids for STAT1, STAT2, and IRF9, as well as Flag-tagged JAK1 and TYK2 plasmids, were purchased from Miaolingbio (Wuhan, China). The ISRE promoter-driven luciferase reporter and pRL-TK internal control plasmids were obtained from Genomeditech (Shanghai, China).

### 4.4. Dual-Luciferase Reporter (DLR) Assay

HEK293T cells were seeded into 24-well plates and grown to approximately 80% confluent. The cells were then transfected with either D345L or empty control vector, together with 125 ng of ISRE-firefly luciferase reporter and 20 ng of pRL-TK Renilla internal control. After 24 h, cells were lysed and dual-luciferase activities were quantified with the TransGen Biotech (Beijing, China) kit following the manufacturer’s instructions. Firefly/Renilla ratios were calculated to yield relative luciferase activity; all transfections were performed in triplicate.

### 4.5. Western Blotting (WB) Analysis

Cell lysates were prepared using radio immunoprecipitation assay (RIPA) lysis buffer containing the phosphatase and protease inhibitor cocktail (Cowin Bio, Nanjing China). Total protein was quantified with the Pierce™ BCA kit (Thermo Fisher Scientific, Waltham, MA, USA). Samples were boiled for 10 min in reducing SDS-PAGE loading buffer (Cowin Bio), resolved on 4–12% ExpressPlus™ gels (GenScript Biotech, Nanjing, China), and transferred to nitrocellulose membranes (Merck, Shanghai China). After blocking with 5% (*w*/*v*) skim milk in TBST (0.05% Tween-20), membranes were probed with primary and secondary antibodies and developed with High-sig ECL substrate (Tanon, Shanghai, China).

### 4.6. RNA Extraction and Quantitative Reverse Transcription Polymerase Chain Reaction

Total RNA was isolated from cultured cells with the RNA Preparation Kit (UElandy, Suzhou, China) and reverse-transcribed using RT Master Mix (TaKaRa, Beijing China). qPCR was performed on an ABI 7900HT system with SYBR Green Master Mix (TOYOBO, Shanghai China), with primers listed in Table 1. Target mRNA levels were normalized to porcine or human GAPDH (as appropriate) and quantified by the 2^(−ΔΔCt)^ method.

### 4.7. Co-Immunoprecipitation (Co-IP) Assays

HEK293T cells seeded in 6-well plates were co-transfected with the indicated plasmids for 24 h. After two washes with ice-cold PBS, cells were lysed on ice in Pierce™ IP Lysis Buffer (Thermo Fisher Scientific) supplemented with phosphatase and protease inhibitor cocktail. Protein A/G magnetic beads (MedChemExpress) were pre-washed three times with binding/wash buffer, then incubated with anti-Flag or anti-HA antibody for 2 h at 4 °C. The antibody-conjugated beads were subsequently mixed with cleared lysates and rotated for 2 h at 4 °C. Immune complexes were washed three times with elution buffer, boiled in reducing SDS-PAGE loading buffer, and analyzed by Western blot.

### 4.8. Confocal Microscopy and Co-Localization Analysis

HEK293T cells seeded on confocal dishes (NEST, Wuxi, China) were transfected with D345L-Flag and STAT1-HA plasmids. After transfection for 24 h, cells were fixed with 4% paraformaldehyde for 30 min at room temperature, permeabilized with 0.1% Triton X-100 for 15 min, and blocked with 5% BSA for 1 h. Following overnight incubation at 4 °C with anti-Flag or anti-HA primary antibodies, cells were stained with Alexa Fluor 594- or 488-conjugated secondary antibodies for 2 h and counterstained with DAPI (Sigma-Aldrich) for 10 min. Images were acquired on a Leica TCS SP8 confocal microscope (Leica Microsystems, Wetzlar, Germany).

### 4.9. Nuclear and Cytoplasmic Extraction

After transfecting HEK-293T cells with the pD345L plasmids, the cells were treated with IFN-β (1000 U/mL) for 8 h after transfection. Nuclear and cytoplasmic fractions were isolated using NE-PER Nuclear and Cytoplasmic Extraction Reagent (Invent Bio Scientific, Shanghai, China). The extracted subcellular fractions were subjected to SDS-PAGE and immunoblotting.

### 4.10. Statistical Analysis

Data are expressed as mean ± SD from three independent experiments. Statistical comparisons were made with one-way ANOVA in GraphPad Prism 5.0. Fluorescence intensity was quantified with ImageJ 1.54g. Significance levels: * *p* < 0.05, ** *p* < 0.01, *** *p* < 0.001, and ns, not significant.

## 5. Conclusions

**Figure 9:**
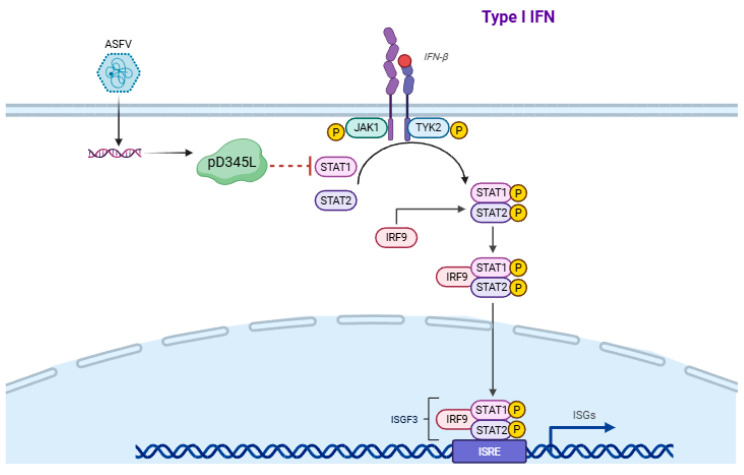
The ASFV pD345L protein interacts with STAT1, degrades STAT1 through the autophagy pathway, and affects the nuclear translocation of STAT1 to inhibit IFN-β signaling, and this inhibitory effect is related to its enzymatic activity.

In summary, we have for the first time demonstrated that the ASFV pD345L is capable of binding to STAT1 through its exonuclease activity, making it a target for autophagy-dependent degradation and preventing its accumulation in the cell nucleus, thereby inhibiting the IFN-β signaling pathway (Figure 9). These findings will provide more knowledge for understanding the mechanism by which ASFV bypasses the host’s innate immune system.

## Figures and Tables

**Table 1:** Detection primer for real-time qPCR.

Primer	Sequences (5′-3′)
VSV-F	TGCAAGGAAAGCATTGAACAA
VSV-R	GAGGAGTCACCTGGACAATCAC
SEV-F	AGCCAGAGGCAGAAGCACA
SEV-R	CTCGGTTTTCATCTTCAGCAC
hGAPDH-F	TCATGACCACAGTCCATGCC
hGAPDH-R	GGATGACCTTGCCCACAGCC
hISG15-F	GCTGGTGGTGGACAAATGC
hISG15-R	ACTTGCTGCTTCAGGTGGG
hISG54-F	CAAAATGAAAGAGCGAAGGTG
hISG54-R	CAATGGCGTTCTGAGATGGT
hDDX58-F	AGAAGAGTACCACTTAAACCCAGAG
hDDX58-R	CCACGTCCAGTCAATATGCC
hOAS1-F	GGCTGAATTACCCATGCTTT
hOAS1-R	AGGCTCGTCTTGTCTGTTGC
SUS-GAPDH-F	TCATCATCACTGCCCCTTCT
SUS-GAPDH-R	GTCATGAGTCCCTCCACGAT
SUS-ISG15-F	GGTGCAAAGCTTCAGAGACC
SUS-ISG15-R	GTCAGCCAGACCTCATAGGC
SUS-ISG54-F	ACAGGAACTAATAGGACACGCTC
SUS-ISG54-R	CACTTGTTTGGCTACAGGAGG
SUS-DDX58-F	CCTGGTTTAGGGACGATGAGG
SUS-DDX58-R	TCCAAAAAGCCTCGGAACCA
SUS-MX1-F	ACAAGATAGTGGACGTGGCG
SUS-MX1-R	TCCCTGAAATGTTCGTGGTT

## Data Availability

All data generated or analyzed during this study are included in this published article.

## References

[1] Lopera-Madrid J., Medina-Magües L.G., Gladue D.P., Borca M.V., Osorio J.E. (2021). Optimization in the Expression of ASFV Proteins for the Development of Subunit Vaccines Using Poxviruses as Delivery Vectors. Sci. Rep..

[2] Gómez-Villamandos J., Bautista M., Sánchez-Cordón P., Carrasco L. (2013). Pathology of African Swine Fever: The Role of Monocyte-Macrophage. Virus Res..

[3] Blome S., Gabriel C., Beer M. (2013). Pathogenesis of African Swine Fever in Domestic Pigs and European Wild Boar. Virus Res..

[4] Ebwanga E.J., Ghogomu S.M., Paeshuyse J. (2021). African Swine Fever in Cameroon: A Review. Pathogens.

[5] Zajac M.D., Trujillo J.D., Yao J., Kumar R., Sangewar N., Lokhandwala S., Sang H., Mallen K., McCall J., Burton L. (2023). Immunization of Pigs with Replication-Incompetent Adenovirus-Vectored African Swine Fever Virus Multi-Antigens Induced Humoral Immune Responses but No Protection Following Contact Challenge. Front. Vet. Sci..

[6] Sánchez-Cordón P.J., Montoya M., Reis A.L., Dixon L.K. (1997). African Swine Fever: A Re-Emerging Viral Disease Threatening the Global Pig Industry. Vet. J..

[7] Zhou X., Li N., Luo Y., Liu Y., Miao F., Chen T., Zhang S., Cao P., Li X., Tian K. (2018). Emergence of African Swine Fever in China, 2018. Transbound. Emerg. Dis..

[8] Alonso C., Borca M., Dixon L., Revilla Y., Rodriguez F., Escribano J.M., ICTV Report Consortium (2018). ICTV Virus Taxonomy Profile: Asfarviridae. J. Gen. Virol..

[9] Wilkinson P. (1984). The Persistence of African Swine Fever in Africa and the Mediterranean. Prev. Vet. Med..

[10] Karger A., Pérez-Núñez D., Urquiza J., Hinojar P., Alonso C., Freitas F.B., Revilla Y., Potier M.-F.L., Montoya M. (2019). An Update on African Swine Fever Virology. Viruses.

[11] Ravilov R., Galeeva A., Frolov G., Efimova M., Zakirova E., Rizvanov A., Hisamutdinov A., Garipov L., Mingaleev D. (2023). Efficient Delivery of the Immunodominant Genes of African Swine Fever Virus by Adeno-Associated Virus Serotype 2. Vet. World.

[12] Vinuela E. (1985). African Swine Fever Virus. Current Topics in Microbiology and Immunology.

[13] Machuka E.M., Juma J., Muigai A.W.T., Amimo J.O., Pelle R., Abworo E.O. (2022). Transcriptome profile of spleen tissues from locally-adapted Kenyan pigs (*Sus scrofa*) experimentally infected with three varying doses of a highly virulent African swine fever virus genotype IX isolate: Ken12/busia.1 (ken-1033). BMC Genom..

[14] Riera E., Pérez-Núñez D., García-Belmonte R., Miorin L., García-Sastre A., Revilla Y. (2021). African Swine Fever Virus Induces STAT1 and STAT2 Degradation to Counteract IFN-I Signaling. Front. Microbiol..

[15] Afonso C.L., Piccone M.E., Zaffuto K.M., Neilan J., Kutish G.F., Lu Z., Balinsky C.A., Gibb T.R., Bean T.J., Zsak L. (2004). African Swine Fever Virus Multigene Family 360 and 530 Genes Affect Host Interferon Response. J. Virol..

[16] Goodbourn S., Randall R.E. (2009). The Regulation of Type I Interferon Production by Paramyxoviruses. J. Interferon Cytokine Res..

[17] Randall R.E., Goodbourn S. (2008). Interferons and Viruses: An Interplay between Induction, Signalling, Antiviral Responses and Virus Countermeasures. J. Gen. Virol..

[18] Wang Y., Song Q., Huang W., Lin Y., Wang X., Wang C., Willard B., Zhao C., Nan J., Holvey-Bates E. (2021). A Virus-Induced Conformational Switch of STAT1-STAT2 Dimers Boosts Antiviral Defenses. Cell Res..

[19] Stark G.R., Darnell J.E. (2012). The JAK-STAT Pathway at Twenty. Immunity.

[20] Ketkar H., Harrison A.G., Graziano V.R., Geng T., Yang L., Vella A.T., Wang P. (2021). UBX Domain Protein 6 Positively Regulates JAK-STAT1/2 Signaling. J. Immunol..

[21] Nan Y., Wu C., Zhang Y.-J. (2017). Interplay between Janus Kinase/Signal Transducer and Activator of Transcription Signaling Activated by Type I Interferons and Viral Antagonism. Front. Immunol..

[22] Dixon L.K., Abrams C.C., Bowick G., Goatley L.C., Kay-Jackson P.C., Chapman D., Liverani E., Nix R., Silk R., Zhang F. (2004). African Swine Fever Virus Proteins Involved in Evading Host Defence Systems. Vet. Immunol. Immunopathol..

[23] Reis A.L., Abrams C.C., Goatley L.C., Netherton C., Chapman D.G., Sanchez-Cordon P., Dixon L.K. (2016). Deletion of African Swine Fever Virus Interferon Inhibitors from the Genome of a Virulent Isolate Reduces Virulence in Domestic Pigs and Induces a Protective Response. Vaccine.

[24] Zsak L., Sur J.H., Burrage T.G., Neilan J.G., Rock D.L. (2001). African Swine Fever Virus (Asfv) Multigene Families 360 and 530 Genes Promote Infected Macrophage Survival. Sci. World J..

[25] Ye G., Liu H., Liu X., Chen W., Li J., Zhao D., Wang G., Feng C., Zhang Z., Zhou Q. (2023). African Swine Fever Virus H240R Protein Inhibits the Production of Type I Interferon through Disrupting the Oligomerization of STING. J. Virol..

[26] Liu E., Li Z., Zhu X., Zhou J., Liu H., Liang C., Chen Y., Qi Y., Wang A. (2025). The African Swine Fever Virus pH108R Protein Negatively Regulates Innate Immune Response by Inhibiting NF-κB Activation. Curr. Microbiol..

[27] Liu X., Ye G., Zeng Y., Wu H., Dong S., He X., Zhou Q., Liu H., Zhang Z., Li J. (2025). African Swine Fever Virus pB318L Suppresses Inflammatory Response by Inhibiting NF-κB Activation and NLRP3 Inflammasome Formation. PLoS Pathog..

[28] Zhao J., Mou C., Zhu L., Sun X., Chen Z. (2025). African Swine Fever Virus-Encoded pE248R Protein Inhibits Interferon Production via Blocking RIG-I-Mediated Antiviral Signaling. Int. J. Biol. Macromol..

[29] Redrejo-Rodríguez M., Rodríguez J.M., Salas J., Salas M.L., Redrejo-Rodríguez M., Rodríguez J.M., Salas J., Salas M.L. (2011). Repair of Viral Genomes by Base Excision Pathways: African Swine Fever Virus as a Paradigm. DNA Repair—On the Pathways to Fixing DNA Damage and Errors.

[30] Chen H., Wang Z., Gao X., Lv J., Hu Y., Jung Y.-S., Zhu S., Wu X., Qian Y., Dai J. (2022). ASFV pD345L Protein Negatively Regulates NF-κB Signalling by Inhibiting IKK Kinase Activity. Vet. Res..

[31] Qureshi S.A., Salditt-Georgieff M., Darnell J.E. (1995). Tyrosine-Phosphorylated Stat1 and Stat2 plus a 48-kDa Protein All Contact DNA in Forming Interferon-Stimulated-Gene Factor 3. Proc. Natl. Acad. Sci. USA.

[32] Cheon H., Holvey-Bates E.G., Schoggins J.W., Forster S., Hertzog P., Imanaka N., Rice C.M., Jackson M.W., Junk D.J., Stark G.R. (2013). IFNβ-dependent Increases in STAT1, STAT2, and IRF9 Mediate Resistance to Viruses and DNA Damage. EMBO J..

[33] Wang D., Chen J., Yu C., Zhu X., Xu S., Fang L., Xiao S. (2019). Porcine Reproductive and Respiratory Syndrome Virus Nsp11 Antagonizes Type I Interferon Signaling by Targeting IRF9. J. Virol..

[34] Li P., Zhu Z., Zhang X., Dang W., Li L., Du X., Zhang M., Wu C., Xue Q., Liu X. (2019). The Nucleoprotein and Phosphoprotein of Peste Des Petits Ruminants Virus Inhibit Interferons Signaling by Blocking the JAK-STAT Pathway. Viruses.

[35] Kovall R., Matthews B.W. (1997). Toroidal Structure of Lambda-Exonuclease. Science.

[36] He W.-R., Yuan J., Ma Y.-H., Zhao C.-Y., Yang Z.-Y., Zhang Y., Han S., Wan B., Zhang G.-P. (2022). Modulation of Host Antiviral Innate Immunity by African Swine Fever Virus: A Review. Animals.

[37] Zhang K., Yang B., Shen C., Zhang T., Hao Y., Zhang D., Liu H., Shi X., Li G., Yang J. (2022). MGF360-9L Is a Major Virulence Factor Associated with the African Swine Fever Virus by Antagonizing the JAK/STAT Signaling Pathway. mBio.

[38] Li M., Liu X., Peng D., Yao M., Wang T., Wang Y., Cao H., Wang Y., Dai J., Luo R. (2024). The I7L Protein of African Swine Fever Virus Is Involved in Viral Pathogenicity by Antagonizing the IFN-γ-Triggered JAK-STAT Signaling Pathway through Inhibiting the Phosphorylation of STAT1. PLoS Pathog..

[39] Li D., Peng J., Wu J., Yi J., Wu P., Qi X., Ren J., Peng G., Duan X., Ru Y. (2023). African Swine Fever Virus MGF-360-10L Is a Novel and Crucial Virulence Factor That Mediates Ubiquitination and Degradation of JAK1 by Recruiting the E3 Ubiquitin Ligase HERC5. mBio.

[40] Chen Q., Wang X.X., Jiang S.W., Gao X.T., Huang S.Y., Liang Y., Jia H., Zhu H.F. (2023). MGF360-12L of ASFV-SY18 Is an Immune-Evasion Protein That Inhibits Host Type I IFN, NF-κB, and JAK/STAT Pathways. Pol. J. Vet. Sci..

[41] Li L., Fu J., Li J., Guo S., Chen Q., Zhang Y., Liu Z., Tan C., Chen H., Wang X. (2022). African Swine Fever Virus pI215L Inhibits Type I Interferon Signaling by Targeting Interferon Regulatory Factor 9 for Autophagic Degradation. J. Virol..

